# Research on Two-Round Self-Balancing Robot SLAM Based on the Gmapping Algorithm

**DOI:** 10.3390/s23052489

**Published:** 2023-02-23

**Authors:** Jianwei Zhao, Jinyu Li, Jiaxin Zhou

**Affiliations:** School of Mechanical Electronic & Information Engineering, China University of Mining and Technology, Beijing 100083, China

**Keywords:** SLAM, two-wheel self-balancing robot, PID controller, Gmapping algorithm

## Abstract

Aiming at the inconvenience of inspection and monitoring of coal mine pump room equipment in a narrow and complex environment, this paper proposes and designs a two-wheel self-balancing inspection robot based on laser SLAM. Using SolidWorks, the three-dimensional mechanical structure of the robot is designed, and the overall structure of the robot is analyzed by finite element statics. The kinematics model of the two-wheel self-balancing robot was established, and the multi-closed-loop PID controller was used to design the two-wheel self-balancing control algorithm of the robot. The 2D LiDAR-based Gmapping algorithm was used to locate the robot and construct the map. Through the self-balancing test and anti-jamming test, it is verified that the self-balancing algorithm designed in this paper has a certain anti-jamming ability and good robustness. By using Gazebo to build a simulation comparison experiment, it is verified that the selection of the particle number is of great significance for improving the map accuracy. The actual test results show that the constructed map has high accuracy.

## 1. Introduction

As the country vigorously renovates and standardizes the safety management of coal mines, it also promotes the development of coal mine enterprises in the direction of digitization and being unmanned. In the electric power and petrochemical industries, various wheeled, tracked, or rail inspection robots have been used in many cases. However, the inspection work of the main drainage system equipment in the coal mine still needs to be completed manually, and there are some problems, such as manual slack, untimely inspection, and inconsistent inspection results. The research and development of the robot applied in the inspection of drainage equipment of coal mine pump room is of great significance to the safety production of coal mine enterprises.

At first, the known map was stored in the inspection robot to realize autonomous positioning and navigation. In recent years, robot positioning and navigation have been mainly realized by setting up rails above the inspection environment [1]. However, this method cannot be changed with the changes in the inspection environment. In order to solve the limitations of this way of working, SLAM (Simultaneous localization and mapping) technology was developed. SLAM technology was first proposed by researchers in the 1980s. After that, algorithms, such as Kalman filtering, extended Kalman filtering, untraced Kalman filtering, particle filtering, Fast-Slam, and Gmapping, were gradually proposed [2].

The two-wheel self-balancing robot has the characteristics of lightweight, flexible movement, and simple structure, which are suitable for movement in a crowded and narrow environment. In recent years, Qiu Jiandu from the Taiyuan University of Technology has studied the inspection robot of a coal mine water pump room optimized after Fast-Slam, but he used a four-wheel robot [3]. In this paper, the two-wheel self-balancing robot is used to integrate environmental perception, autonomous decision-making, and control execution. The structure has the characteristics of long durability, small size, flexible steering, and wide application range, which is a more suitable choice for the inspection of the groundwater pump room. The fuzzy PID control-based two-wheel self-balancing robot designed by Qiao Lin from Harbin Engineering University can realize self-balancing quickly, but Qiao Lin does not use the SLAM algorithm for synchronous localization and map construction [4].

Based on the current research hotspot of mobile robots, this paper designs and implements a two-round inspection robot based on laser SLAM, aiming at the inconvenience of equipment detection and monitoring in a coal mine pump room, a narrow and complex environment. The robot has functions such as self-balancing movement, precise positioning, and map construction. The robot can accurately locate and construct the environment map through the laser radar sensor, which can support real-time data transmission during the inspection process to facilitate the maintenance and management of the operator. The research content of this paper is of great significance for improving the efficiency of indoor inspection and ensuring workers’ safety.

## 2. Design of a Self-Balancing Mobile System for the Inspection Robot

### 2.1. Overall Mechanical Structure Design of the Inspection Robot

**Figure 1:**
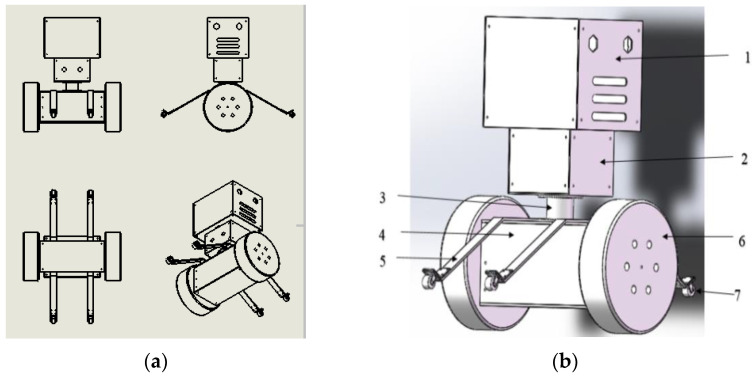
(**a**) Two-dimensional plan of inspection robot. (**b**) Three-dimensional structure drawing of inspection robot.

The overall structure of the inspection robot adopts a tower structure design, which is composed of seven parts: head structure 1, trunk structure 2, waist structure 3, bottom structure 4, protection bracket 5, wheel 6, and guide wheel 7, from top to bottom. The structure of each part is connected and fixed by screws, and the whole inspection robot is formed by processing and assembly. The robot adopts large chassis width; the facade is small at the top and large at the bottom. The design method has a compact structure, large stiffness, and small wind resistance. In order to facilitate assembly and disassembly, the overall structure adopts an axisymmetric design, with each part of the modular design easy to replace and update. The overall structure design of the inspection robot is a rigid connection. Figure 1a shows the 2D plan of the inspection robot, and Figure 1b shows the 3D structure of the inspection robot.

### 2.2. Statics Analysis of Two-Wheel Self-Balancing Mechanical Structures

The 3D structure model of the inspection robot was introduced into the Simulation toolbox of SolidWorks for simulation analysis.

Finite element analysis usually includes three basic steps: 1. Pre-processing; 2. Solve the problem. 3. Post-processing. The pre-processing includes the processing model, defining analysis type, adding material attributes, and applying load and meshes [5]. Post-processing is mainly used to analyze the results and obtain the required force results.

Firstly, the finite element model needs to be established, and the solid model is constructed by the 3D structural design software. Then, the solid model is meshed, the nodes and elements are generated according to the size of the meshed, and the modeling of the finite element model is completed. Statics analysis can verify whether the stiffness and fatigue strength meet the design requirements. By analyzing the simulation results, the stress distribution of each part of the inspection robot under the maximum load can be determined.

The overall structural materials of the inspection robot are mainly the T300 carbon fiber plate and the Q235 carbon steel. In the Simulation software, there is no material parameter of Q235 carbon steel in the material attribute list. Since the properties of 1023 carbon steel and Q235 are similar, 1023 carbon steel is used as the simulation material in the statics simulation. The properties of 1023 carbon steel are shown in Table 1 below.

T300 carbon fiber plate is mainly composed of carbon fiber and epoxy resin. The material properties of carbon fiber plate are shown in Table 2 in the statics simulation.

#### 2.2.1. Force Analysis of Head Structure of Two-Wheel Self-Balancing Robot

**Figure 2:**
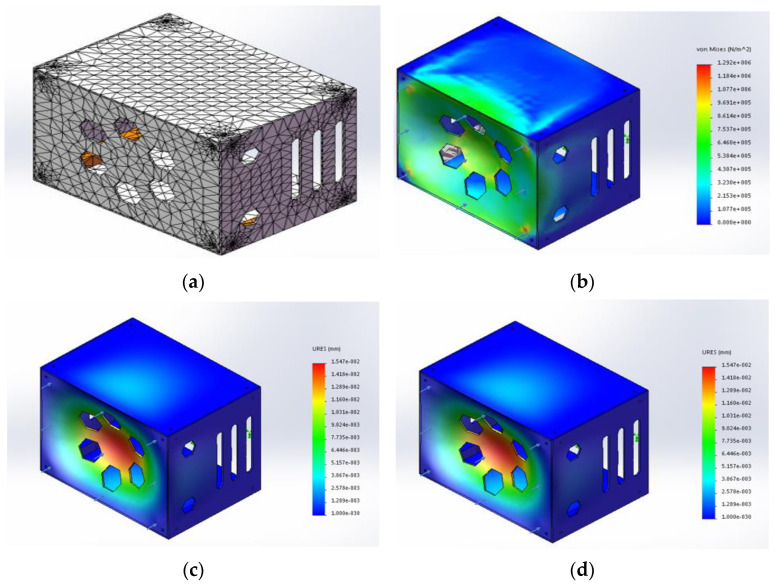
(**a**) Head structure meshing. (**b**) Equivalent stress cloud diagram of head structure. (**c**) Equivalent displacement cloud diagram of head structure. (**d**) Equivalent displacement cloud diagram of head structure.

The upper cover plate of the head structure is mainly equipped with sensors and heavy loads, which is the main force part. In the statics simulation of the head structure, T300 carbon fiber composite material was added as the material property. The external force was loaded on the top cover plate, and the force was set to 50 N. After the basic parameters of the material were set, the head structure was meshed, the mixed meshes were used, and the Jacobi point was set to 4 points. The head structure finite element model, after the meshing operation, is shown in Figure 2a. A total of 18,374 units and 38,038 nodes were obtained. The meshes of each part are of good quality and can meet the requirements of statics. After obtaining the finite element model, the equivalent stress, equivalent displacement, and equivalent strain can be solved. The solution results were processed to obtain the equivalent stress cloud diagram of the head structure, as shown in Figure 2b. The equivalent displacement cloud diagram is shown in Figure 2c. The equivalent strain cloud diagram is shown in Figure 2d.

As can be seen from the equivalent stress cloud diagram of the head structure in Figure 2b, the color depth of the cloud diagram represents the degree of stress, and the deeper the color, the more concentrated the stress. Under the action of 50 N load, it can be seen from Table 3 that the maximum stress of the head structure is 1.29208 × 10^6^ N/m^2^, and the minimum stress is 0 N/m^2^, which is far below the standard strength of material deformation. As can be seen from the equivalent displacement cloud diagram of the head structure in Figure 2c, the color depth of the cloud image represents the displacement, and the deeper the color, the greater the displacement. The maximum displacement is 1.54699 × 10^−2^ mm, and the minimum displacement is 0 mm. The position where the maximum displacement occurs is the center of the upper cover plate, and the position where the minimum displacement occurs is the surrounding edge of the upper cover plate. The displacement value gradually increases from the surrounding to the middle, the overall deformation of the parts is relatively small, and the influence on the strength of the robot can be ignored. According to the equivalent strain cloud diagram of the head structure in Figure 2d, the maximum strain is 7.60008 × 10^−6^, and the minimum strain is 0. Based on the analysis of the above parameters, the design of the head structure is relatively stable, and the stiffness and strength meet the design requirements.

#### 2.2.2. Force Analysis of Bottom Structure of Two-Wheel Self-Balancing Robot

**Figure 3:**
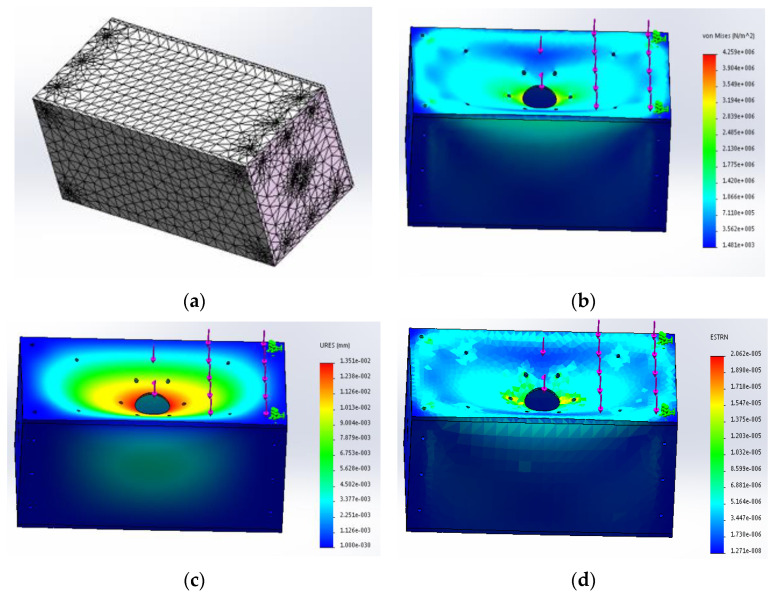
(**a**) Bottom structure meshing. (**b**) Equivalent stress cloud diagram of bottom structure. (**c**) Equivalent displacement cloud map of bottom structure. (**d**) Equivalent strain cloud diagram of bottom structure.

The bottom structure is the core part of the robot. In order to increase the stability and load-bearing performance of the bottom, the bottom structure is made of a 5 mm carbon fiber plate. Compared with other parts of the structure, the bottom of the structure is thickened, which is also convenient for the subsequent addition of expansion modules. In the statics simulation of the bottom structure, the main bearing part is the upper cover plate. In order to verify the bearing capacity of the bottom, the external load is 300 N. After completing the above steps, the bottom structure is meshed to obtain the finite element model of the bottom structure, as shown in Figure 3a. After reasonable grid division, 41,515 units and 72,613 nodes are obtained. After the meshes of the bottom structure are properly divided, the quality of the generated meshes meets the requirements of the statics. After the static simulation, the static equivalent cloud map is solved. The equivalent stress nephogram of the bottom structure is shown in Figure 3b, the equivalent displacement nephogram of the bottom structure is shown in Figure 3c, and the equivalent strain nephogram of the bottom structure is shown in Figure 3d.

According to the equivalent stress cloud diagram of the bottom structure in Figure 3b, the stress of the bottom structure is concentrated in the center and gradually decreases from inside to outside. According to parameter Table 4, the maximum stress of the bottom structure is 4.2585 × 10^6^ N/m^2^, and the minimum stress is 1.48067 × 10^3^ N/m^2^. The deformation strength of the material is far less than that of the standard structure with material properties. As can be seen from the equivalent displacement cloud diagram of the bottom structure in Figure 3c, the displacement position is mainly in the center of the bottom structure, and the displacement size gradually decreases from inside to outside. The maximum displacement is 1.35066 × 10^−2^ mm, and the minimum displacement is 0 mm. The deformation displacement is relatively small, which does not affect the stability of the bottom structure. According to the equivalent strain cloud diagram of the bottom structure in Figure 3d, the maximum strain is 2.06188 × 10^−5^, and the minimum strain is 1.27093 × 10^−8^. Through the above analysis of statics parameters, the design of the bottom structure is reasonable, with good strength and stiffness, certain stability, and load-bearing capacity, meeting the design requirements.

The finite element analysis software SolidWorks Simulation is used to analyze the statics of the inspection robot. By applying the corresponding force to the structure, the equivalent stress cloud diagram, equivalent displacement cloud diagram, and equivalent strain cloud diagram of the structure are obtained, which verifies that the involved structure meets the requirements of working strength and stiffness. Through the analysis results of each structure, it can be seen that each key component has a small shape variable, and the maximum stress is less than the strength requirement of the material itself, which indicates that the structure designed in this paper is reasonable, and the structural stiffness and strength meet the design requirements.

## 3. Modeling and Analysis of the Two-Wheel Self-Balancing Robot System

**Figure 4:**
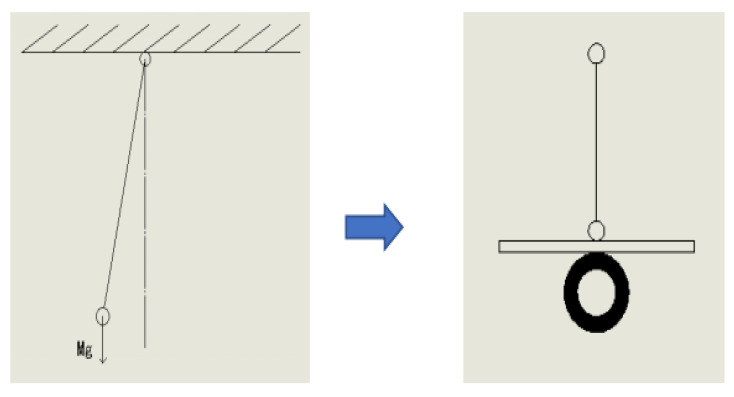
The car body is simplified to an inverted pendulum model.

In the gravity field, thin lines are used to hang heavy objects, which are simplified to form an idealized simple pendulum model [6]. The upright car body can be regarded as an inverted pendulum on the left and right moving platforms, as shown in Figure 4. There are generally two kinds of mathematical models for the inverted pendulum control system, namely, the Newton–Euler method and the Lagrange method [7]. The Newton–Euler method can be used for the first-level linear inverted pendulum model. Firstly, the force analysis of the car and the pendulum rod is carried out, and their equations of motion are obtained, respectively, then the Laplace transform is carried out, and finally, the state space equation of the system can be obtained after sorting.

### 3.1. Force Analysis of the Two-Wheel Self-Balancing Model

**Figure 5:**
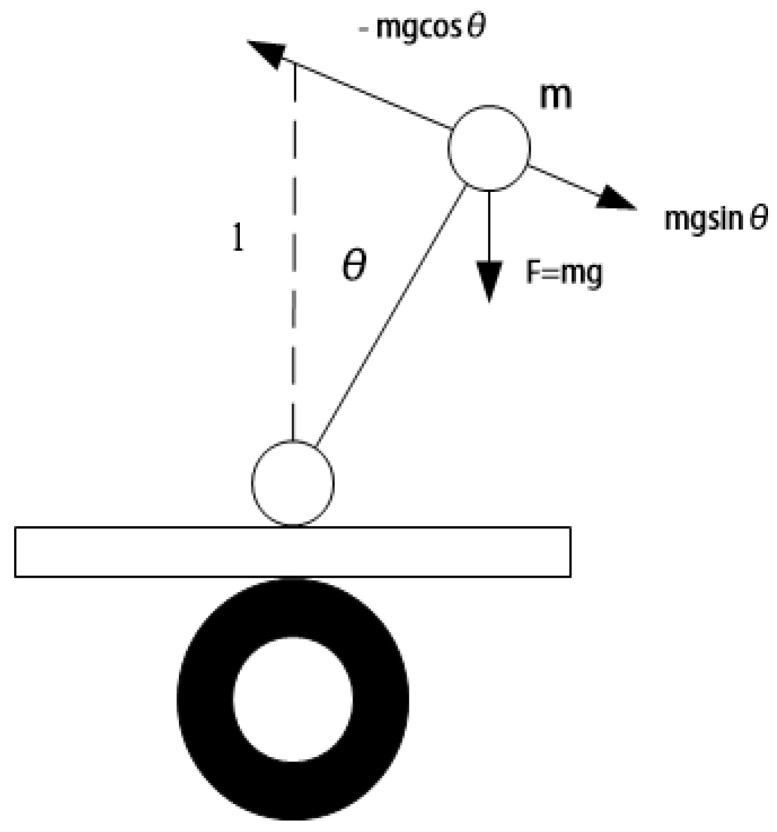
Body force analysis.

When the inverted pendulum is placed out of balance, the recovery force is in the same direction as the displacement, so the inverted pendulum will accelerate away from the vertical position until it falls, so it cannot be stabilized in the vertical position. There are two ways to solve this problem: one is to change the direction of gravity, and the other is to apply extra force so that the restoring force is in the opposite direction of the displacement. Obviously, only the second method is chosen, which controls the bottom wheel of the inverted pendulum to make the car body accelerate. Through the analysis of the inverted pendulum force, we know that it will be subjected to additional inertial force; the force is opposite to the wheel direction; the size is proportional. Force analysis is shown in Figure 5.

According to the body force analysis diagram, when the center of gravity of the inverted pendulum changes, in order to maintain balance in the vertical position of the inverted pendulum, it is necessary to control the bottom wheel to accelerate to the right. The pendulum is balanced by adding extra force and speed so that the restoring force is in the opposite direction of the wheel displacement. The restoring force of the inverted pendulum is as follows:

(1) F=mgsinθ−macosθ

In the above formula, *m* is the total mass of the inspection robot, *g* is the acceleration of gravity, and the direction is vertically downward, *a* is wheel acceleration, direction to the right, and θ is the body tilt angle. Because the inclination angle of the car body θ is small, according to the trigonometric function, it can be linearized. It is assumed that under the negative feedback control, the wheel acceleration a is proportional to the inclination angle of the body θ, and the proportional coefficient is denoted as $k_{1}$, and $k_{1}>g$, Equation (1) can be changed into:

(2) $$F=mg\sin\theta -ma\cos\theta \approx mg\theta -k_{1}\theta$$

In addition, in order to make the inverted pendulum system quickly reach the stable state in the vertical position, it is necessary to increase the control damping force of the system. At the same time, the control damping force should be proportional to the inclination angle of the body, in the opposite direction.

(3) $$F=mg\theta -mk_{1}\theta -mk_{2}\frac{d\theta }{dt}$$

Using the above method, the inverted pendulum model is transformed into a simple pendulum model, which can be stabilized in the vertical position. According to Equations (1) and (3), the wheel acceleration control algorithm is shown as follows:

(4) $$a=k_{1}\theta +k_{2}\frac{d\theta }{dt},$$

where $k_{2}$ is the proportion coefficient and $k_{2}>0$; $\frac{d\theta }{dt}$ is the vehicle body inclination angle speed; the wheel acceleration can be controlled by superposition of angle and angular velocity. In Equation (4) above, the proportion coefficient of the tilt angle $k_{1}$ determines whether the body can maintain balance in the vertical position; the proportional coefficient of inclination angle velocity $k_{2}$ determines whether the vehicle body is quickly stabilized in the vertical position and plays a role in suppressing the system vibration.

### 3.2. Modeling and Analysis of the Two-Wheel Self-Balancing Robot System

**Figure 6:**
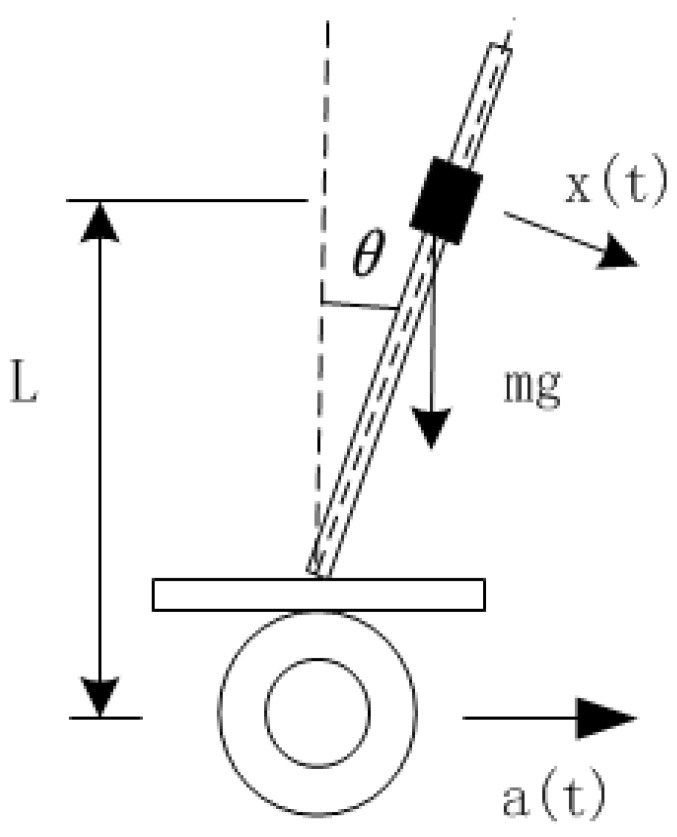
Force analysis of robot model.

As the whole robot is connected by a rigid structure, the system structure model of the inspection robot is established, as shown in Figure 6, where *L* is the distance between the center of gravity of the body and the wheel axis is the tilt angle of the robot, and the mass of the body is m.

Assuming that the angular acceleration x(t) is generated under the action of external force and the acceleration a(t) generated by the wheel, the force analysis is carried out on the robot model to obtain the motion model of the inspection robot:

(5) $$L\frac{d^{2}\theta (t)}{dt^{2}}=g\sin[\theta (t)]-a(t)\cos[\theta (t)]+Lx(t)$$

In the range of small angles, the equation of motion can be linearized:

(6) $$L\frac{d^{2}\theta (t)}{dt^{2}}=g\theta (t)-a(t)+Lx(t)$$

When the vehicle body is in a stable equilibrium state in the vertical position, and the inclination angle θ(t) is 0, then the equation becomes:

(7) $$L\frac{d^{2}\theta (t)}{dt^{2}}=g\theta (t)+Lx(t)$$

By transforming Equation (7), the system input–output transfer function is:

(8) $$H(s)=\frac{\theta (s)}{X(s)}=\frac{1}{s^{2}-\frac{g}{L}}$$

According to the transfer function, the system has two poles, as shown below:

(9) $$s_{p}=\pm \sqrt{\frac{g}{L}}$$

**Figure 7:**
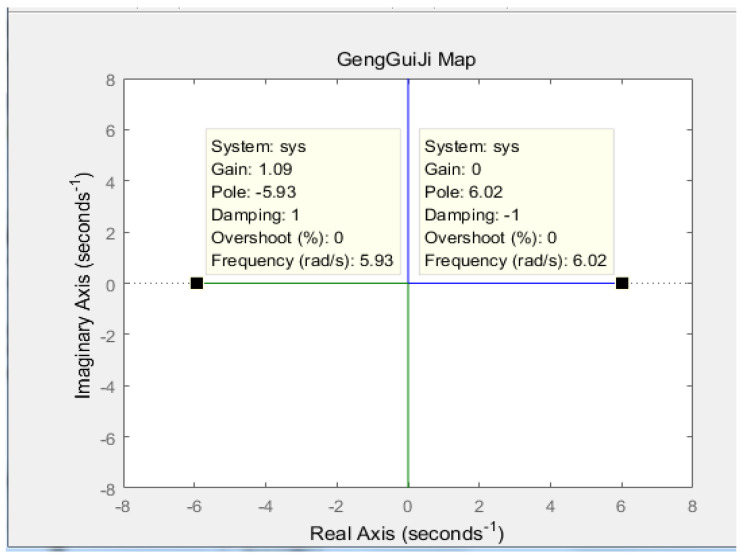
The original root locus of the robot.

According to Equation (8), MATLAB is used to draw the root trajectory diagram of the transfer function, where the gravity acceleration g is 9.8 m/s^2^, and the center of gravity *L* is 0.27 m. The result is shown in Figure 7 below.

**Figure 8:**
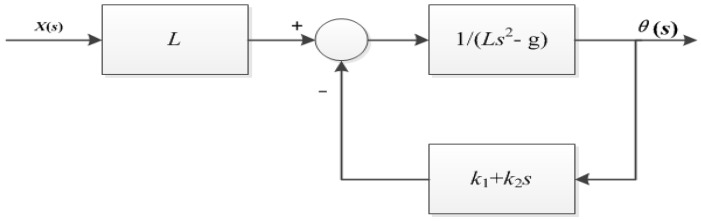
System block diagram of adding negative feedback control.

As can be seen from Figure 7, one pole of the system is located in the right half plane of the system, and the system is in an unstable state. Therefore, the system needs to introduce negative feedback control, as shown in Figure 8 below.

After adding proportional control and differential control, the transfer function of the system is

(10) $$H(s)=\frac{\theta (s)}{X(s)}=\frac{1}{s^{2}+\frac{k_{2}}{L}s+\frac{k_{1}-g}{L}}$$

Then, the pole of the transfer function is

(11) $$s_{p}=\frac{-k_{2}\pm \sqrt{k_{2}-4L(k_{1}-g)}}{2L}$$

**Figure 9:**
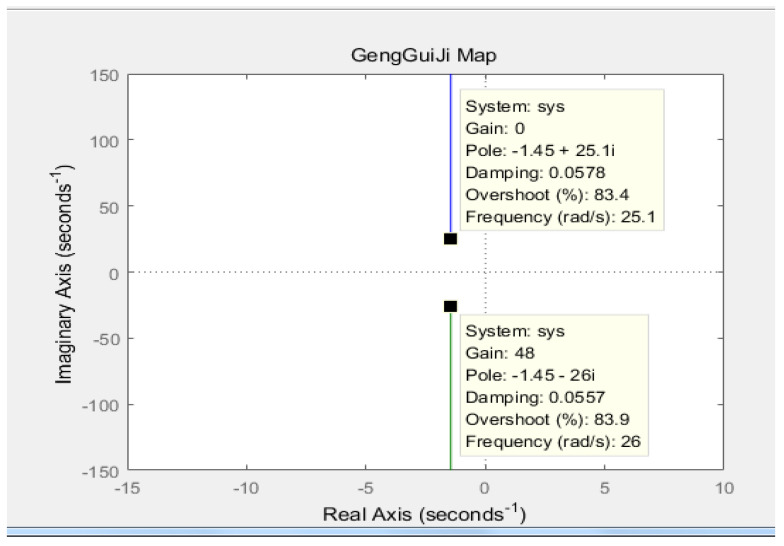
Improved root locus diagram.

MATLAB was used to draw the improved root trace diagram of the transfer function, as shown in Figure 9 below:

When *k*_1_ > *g* and *k*_2_ > 0, it can be seen from the root trajectory diagram that both poles of the system are in the left half plane of the S-plane, and the system is in a stable state.

### 3.3. Design of the Self-Balancing Algorithm for the Two-Wheel Robot

**Figure 10:**
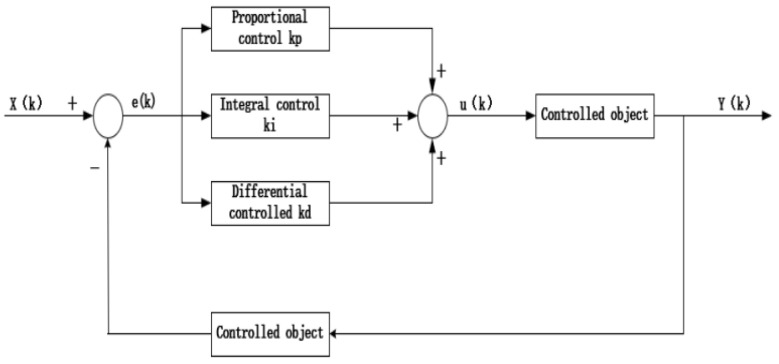
PID controller block diagram.

By analyzing the mathematical model of the inspection robot, it can be seen that one pole of the system is in the right part of the S-plane, and the system is in an unstable state when there is no external force. Therefore, additional controllers are needed to ensure the stable operation of the robot. At present, automatic control technology is based on the core idea of feedback; the feedback process mainly includes three parts—measurement, control, and comparison. The actual parameter value of the controlled object is collected by the sensor and compared with the set expected value. The error between the expected value and the actual value is used to correct the response of the control system. This paper adopts a PID controller, which is mainly composed of three parts, Proportional unit, Integral unit, and Differential unit. The control block diagram is shown in Figure 10 below.

In the above diagram, *X*(*k*) is the expected value set by the system, *Y*(*k*) is the actual output value of the controlled object, and *e*(*k*) is the deviation value between the expected value *X*(*k*) and the actual value *Y*(*k*), which is taken as the input value of the PID controller. After adjusting the deviation parameters, the output of the PID controller *u*(*k*) is the input quantity of the controlled image. After adjusting the parameters of the PID controller, the error caused by the accumulation of the controlled object can be effectively reduced, and the reliability and fast response of the system can be improved. The PID controller algorithm is shown as follows:

(12) $$u(k)=K_{P}\left[e(k)+\frac{T}{T_{I}}\sum_{j=0}^{k}e(j)+\frac{TD}{T}(e(k)-e(k-1))\right]$$

In Equation (12), *K_P_* represents the proportion coefficient, *T* represents the sampling period, and *e*(*k*) represents the input error value at the time of sampling. Moreover, *e*(*k* − 1) represents the sampling error at the time (*k* − 1). The function of the proportional term *K_P_* is to improve the steady-state accuracy of the system and speed up the response accuracy.

At present, the common PID controller algorithms are incremental PID and digital PID [8]. Because the digital incremental PID controller only adjusts the current error as a controlled quantity, it is a recursive algorithm. The algorithm requires less computation and can improve the reliability of the system. This algorithm is chosen as the motor control strategy in this paper, and its control algorithm is shown in Equation (13).

(13) $$\begin{array}{ll} \Delta u(k) & =u(k)-u(k-1)\\ & =K_{P}(e(k)-e(k-1))+K_{I}e(k)+\\ & \;K_{D}(e(k)-2e(k-1)+e(k-2)) \end{array}$$

In Equation (13), *K_I_* denotes the integral coefficient, *K_D_* is the differential coefficient, the integral term *K_I_* is used to eliminate the steady-state error and improve the system’s accuracy, the differential term *K_D_* reduces system oscillation and prevents system overshoot. Where *u*(*k*) and *u*(*k* − 1) represent the output values of the PID controller at adjacent moments, and *e*(*k*), *e*(*k* − 1), and *e*(*k* − 2) represent the deviation between the expected value and the actual value at three adjacent moments. The incremental PID controller can obtain the control output at the next moment by using these five parameter values. In practical application, the algorithm structure can be flexibly changed according to the characteristics and control requirements of the controlled object. The commonly used controller structure includes the P controller, PI controller, PD controller, PID controller, and so on.

#### 3.3.1. Two-Wheel Self-Balancing Robot Upright Ring Control

**Figure 11:**
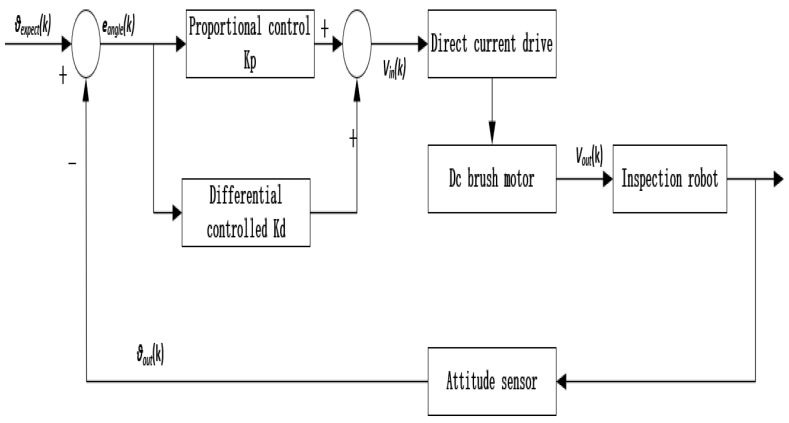
Upright ring control block diagram.

According to the above mathematical model of the robot, the precondition for the robot to maintain self-balance is to obtain the attitude data of the vehicle body, and the controller calculates the appropriate motor input speed through the attitude angle information so that the vehicle body can maintain balance. In the upright balance control, the attitude information of the vehicle body is collected by the MPU6050 and processed by the STM32 controller to output the desired attitude angle *θexpect*(*k*) required by the control. The desired speed control *Vin*(*k*) is obtained after the calculation of the Angle PD controller. The control quantity sends instructions to the DC driver to drive the DC brush motor to rotate, and the robot moves with speed *Vout*(*k*), resulting in the change of the actual attitude angle of the robot. The actual attitude angle *θout*(*k*) is collected by the MPU6050 attitude sensor, and the result and the expected attitude angle *θexpect*(*k*) are used as the input of an angle control, which finally keeps the robot in dynamic balance. The control block diagram of the upright ring is shown in Figure 11 below.

**Figure 12:**
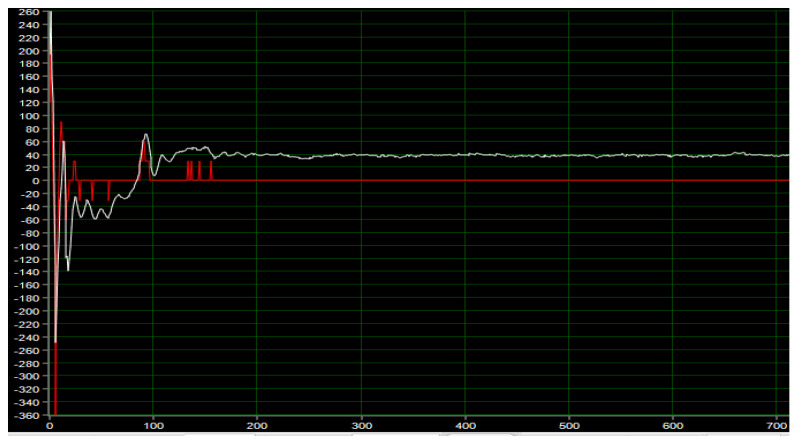
Vertical ring motor test.

The Angle PD controller is applied to the control algorithm of the inspection robot to supply power to the inspection robot and collect the actual and expected speed values of the motor, as shown in Figure 12 below, where the red curve represents the actual speed curve of the motor, the white curve represents the expected speed curve, and the expected speed value is output by the Angle PD controller. As can be seen from the figure, under the action of the Angle PD controller, the motor reaches a stable state within 150 ms, the expected speed converges, the actual output speed is 0, and the robot maintains dynamic balance. Since only a single closed-loop PD controller is used, there is some deviation between the desired and actual speeds, and the robot remains upright near the equilibrium point.

#### 3.3.2. Two-Wheel Self-Balancing Robot Speed Loop Control

**Figure 13:**
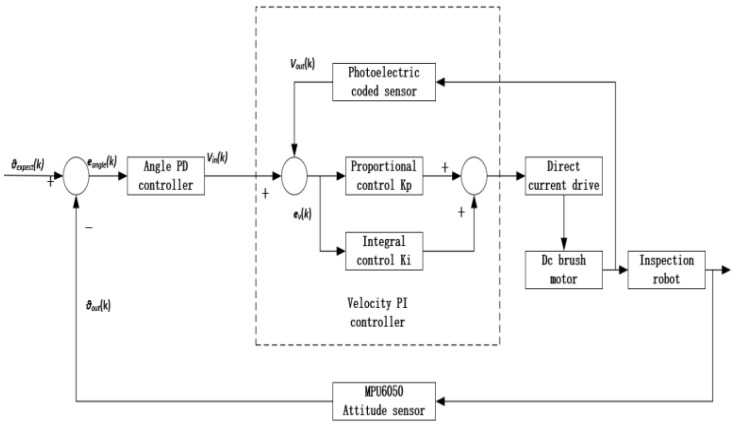
Block diagram of dual closed-loop PID controller.

The control of the upright ring can only allow the robot to maintain self-balance. To control the movement of the vehicle body, it is necessary to increase the speed PI controller, as shown in Figure 13. The actual output speed *Vout*(*k*) is calculated by using the encoder to collect the position information of the motor, and the control deviation *ev*(*k*) is obtained by comparing it with the desired speed value *Vin*(*k*) of the motor. Then the deviation is proportional and integral, adjusted by the feedback input so that the deviation tends to zero. The function of the speed PI controller is to control the actual speed to follow the expected value change, achieve no static error, adjust the dynamic structure of the object, and speed up the dynamic response of the system through closed-loop control to make the system achieve a certain anti-interference ability.

**Figure 14:**
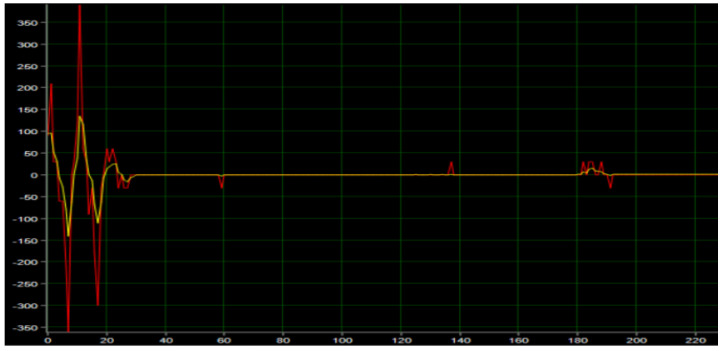
Speed loop test.

The test of the speed loop is shown in Figure 14 below, where the red curve represents the actual speed of the motor, and the yellow curve represents the expected speed, which is output by the speed PI controller. As can be seen from the figure, when the motor is powered, the speed is adjusted by a small amplitude of oscillation and reaches a stable state after 30 ms. The expected speed and the actual speed converge rapidly, and the expected speed can track the actual speed well. It is verified that the speed PI controller has a fast dynamic response ability.

## 4. Research on Two Rounds of the Self-Balancing Robotic LiDAR SLAM

### 4.1. Two Rounds of the Self-Balancing Laser SLAM Research

Simultaneous Localization and Mapping (SLAM) of autonomous mobile robots in indoor environments is a research hotspot in the field of mobile robots [9]. With the continuous update and development of SLAM technology, more and more attention has been paid to technology in this field in recent years. Wu Yuehua et al. [10] from Tianjin University obtained the corresponding SLAM algorithm based on sound waves based on the environmental information provided by sonar sensors and achieved good research results. At present, according to the different ways in which the SLAM algorithm applies sensors to obtain data, it can be divided into laser SLAM [11], which uses LiDAR as the sensor, and visual SLAM, which uses a camera as the sensor [12]. The LiDAR sensor has the advantages of low manufacturing cost, simple use, and convenient maintenance. The technology based on laser SLAM is relatively mature and stable. In the inspection process, the robot can explore the unknown environment, realize the construction of the environment map, and finally, realize the autonomous mobile inspection function. Considering the above considerations, the navigation method adopted in this paper is the laser SLAM technology.

### 4.2. Two-Wheel Self-Balancing Robotic LiDAR Sensor

**Figure 15:**
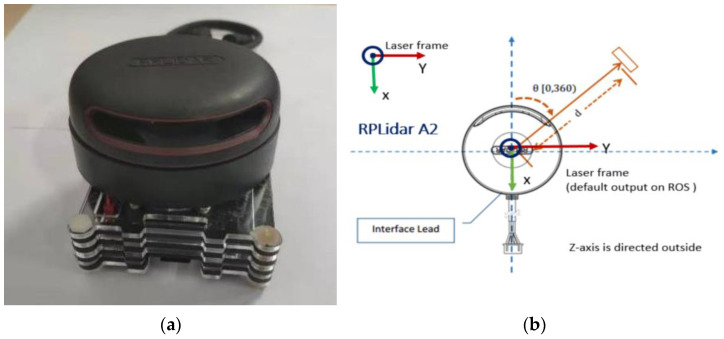
(**a**) Silan RPLIDAR A2 LiDAR. (**b**) Working principle of RPLiDAR A2.

The LiDAR sensor selects the RPLIDAR A2 radar of Slan Technology as the core sensor of the mobile robot in this project, as shown in Figure 15a. When working, the ranging core of RPLIDAR A2 will rotate clockwise to realize the 360-degree omnidirectional scanning ranging detection of the surrounding environment, so as to obtain the two-dimensional contour map of the surrounding environment [13], as shown in Figure 15b.

The laser emitter and CMOS image sensor are integrated into one device. The distance of the obstacles around the sensor can be obtained by 360 degrees of the constant rotation of the motor on the LiDAR. After obtaining the range value, it is also necessary to know the exact angle corresponding to the range value. The rotation angle can be directly obtained by the photoelectric encoder installed on the LiDAR. The LiDAR rotates 360 degrees by driving a brushless motor. Each rotation of the motor triggers a numerical change in the photoelectric encoder and then triggers a collection by the laser receiver. In this way, the range value of each angle can be accurately obtained. These collected distance and angle values are processed as the basis for further research on robot localization and the construction of an environment map.

### 4.3. Research on the Gmapping Algorithm of the Two-Wheel Self-Balancing Robot Based on the 2D LiDAR

In the problem of robot localization, it is necessary to estimate the position and attitude of the robot. Through continuous movement and observation, the robot can obtain information about the surrounding environment, gradually increase the information describing its own posture, and finally, get accurate positioning results [14]. The SLAM process can be described by the conditional joint probability distribution $p(x_{1:t},m|z_{1:t},u_{1:t-1})$; that is, the position, attitude, and environment map can be inferred simultaneously from the sensor observation data and motion control data. According to the probability theory, joint probability can be converted into conditional probability, as shown in Equation (14):

(14) P(x,y)=p(y|x)p(x)

Since it is difficult to solve the joint distribution of two variables at the same time, it is optional to solve one of the variables and then take the solution result as a known condition to solve the other variable. By decomposing the problem one by one, the calculation process is simplified, and the difficulty of solving them is greatly reduced.

#### 4.3.1. Particle Filter

Particle Filter is a non-parametric implementation of Bayesian filtering, the purpose of which is to use a large number of weighted random samples and approximate posterior probability density function and to approximate the fitting integral by means of summation [15]. The observation model and system model are used in the process of particle filter operation, and the internal state information of the system can be approximately estimated under a dynamic environment by the data value of observation noise. When updating the observation information, it is necessary to use Bayesian recursive filter for estimation processing, which is mainly divided into two steps. The first step is the prediction (prior to the probability density), as follows:

(15) $$p(x_{k}|Z_{k-1})=\int p(x_{k}|x_{k-1})p(x_{k-1}|Z_{k-1})dx_{k-1}$$

The second step is to update (the posterior probability density) as follows:

(16) $$p(x_{k}|Z_{k})=\int p(z_{k}|x_{k})p(x_{k}|Z_{k-1})d(z_{k}|Z_{k-1}),$$

where *x_k_* is the state sequence representation at time *k*; that is, *x_k_* = {*x*_1_, *x*_2_,…, *x_k_*}, *Z_k_* is the measurement sequence; that is, *Z_k_* = {*z*_1_, *z*_2_,…, *z_k_*}. *p*(*x_k_*|*x*_*k*−1_) is the state of the prior distribution, *p*(*x_k_*|*z*_*k*−1_) is the (*k* − 1) time known probability distribution, *p*(*z_k_*|*z*_*k*−1_) is the normalization constant.

The prediction results are modified by the particle filter, and the SLAM algorithm based on the particle filter effectively solves the problems of high algorithm complexity and low filtering accuracy. This scheme combines the advantages of particle filter and extended Kalman and improves the calculation accuracy of the system while preserving the control robustness of the system.

#### 4.3.2. The Gmapping Algorithm Based on RBPF

The Gmapping algorithm is a SLAM algorithm based on 2D LiDAR that uses the Rao-Blackwellized Particle Filters (RBPF) algorithm to complete the construction of 2D raster maps [16]. The Gmapping algorithm adopts the Rao–Blackwellized particle filter algorithm to divide the SLAM problem into two steps, namely, the localization problem and the map construction problem [17]. Firstly, the self-localization problem is determined, and then, the map is constructed. Rao–Blackwellized particle filter algorithm is used for robot localization, and an extended Kalman filter (EKF) is used for the map construction. The Gmapping algorithm process is the same. At the same time, it is relatively difficult to locate and construct the map. Therefore, it is relatively simple to solve the robot pose first and then construct the map through the pose. After the localization and map construction, the conditional joint probability distribution can be changed into

(17) $$p(x_{1:t},m|z_{1:t},u_{1:t-1})=p(m|x_{1:t},z_{1:t})\cdot p(x_{1:t}|z_{1:t},u_{1:t-1}),$$

where the above equation converts the SLAM problem into the form of the product of two independent posterior probabilities [18]. The running steps of the Gmapping algorithm are mainly divided into four steps: sampling; calculating weight; resampling; and estimating map. Each particle in the algorithm independently records a possible robot trajectory and the corresponding environment map.

The Gmapping algorithm is improved on the basis of the particle filter algorithm, which mainly improves two aspects, namely, selective resampling and improved proposal distribution. Firstly, the resampling step is an important factor affecting the performance of the particle filter. For robot pose estimation, the particle filtering algorithm requires constant particle iteration and resampling to estimate the pose at each moment, and frequent resampling will usually lead to low-weight particles being replaced by high-weight samples, resulting in the particle degradation phenomenon. Resampling is important because the number of particles close to the target distribution is limited. To solve this problem, the Gmapping algorithm calculates the weight of each particle to filter particles before resampling, reduces the number of resampling times, and sets a threshold for the particle weight. Resampling is carried out only when the particle weight is greater than the set threshold. In this way, the sampling times of the algorithm can be reduced, and the particle degradation can be alleviated. The effective particle number is usually used to evaluate the degradation degree of particle weight, as shown below:

(18) $$N_{eff}=\frac{1}{\sum_{i=1}^{N}{\left(w^{\left(i\right)}\right)}^{2}}$$

Secondly, the improved proposal distribution is used to approximate the target distribution in the Gmapping algorithm. After the particle sampling is completed, the observation information at the latest update time is used to weigh the sampled particles, and the particle with a larger value is used to simulate the improved proposal distribution, as shown in Equation (19):

(19) $$p(x_{t}|m_{t-1}^{\left(i\right)},x_{t-1}^{\left(i\right)},z_{t},u_{t-1})=\frac{p(z_{t}|m_{t-1}^{\left(i\right)},x_{t})p(x_{t}|x_{t-1}^{\left(i\right)},u_{t-1})}{p(z_{t}|m_{t-1}^{\left(i\right)},x_{t-1}^{\left(i\right)},u_{t-1})}$$

The Gmapping algorithm is based on RBPF. Firstly, it estimates the trajectory of the robot; then, it builds the environment map according to the trajectory of the robot, and finally, it navigates the robot according to the constructed environment map [19].

### 4.4. A Simulation Experiment of the Inspection Robot Based on the Gmapping Algorithm

In this paper, the ubuntu16.04 system was used as the simulation platform to conduct simulation under ROS environment, and Gazebo was used to build simulation experiments. ROS (Robot Operating System), as an open-source robot development platform, adopts a highly flexible distributed robot architecture, integrates a large number of algorithm models and open-source code, and adopts modular writing rules to simplify the robot development process and difficulty [20]. ROS robot platform is conducive to the rapid testing and algorithm iteration of complex robot systems. Gazebo is a powerful open-source simulation platform that defines a variety of visual entities and can realize dynamic simulation and kinematic simulation, etc. [21].

**Figure 16:**
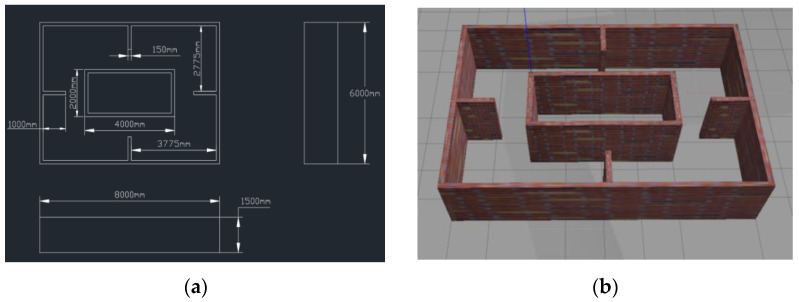
(**a**) Two-dimensional map of simulation map. (**b**) Virtual simulation 3D map.

**Figure 17:**
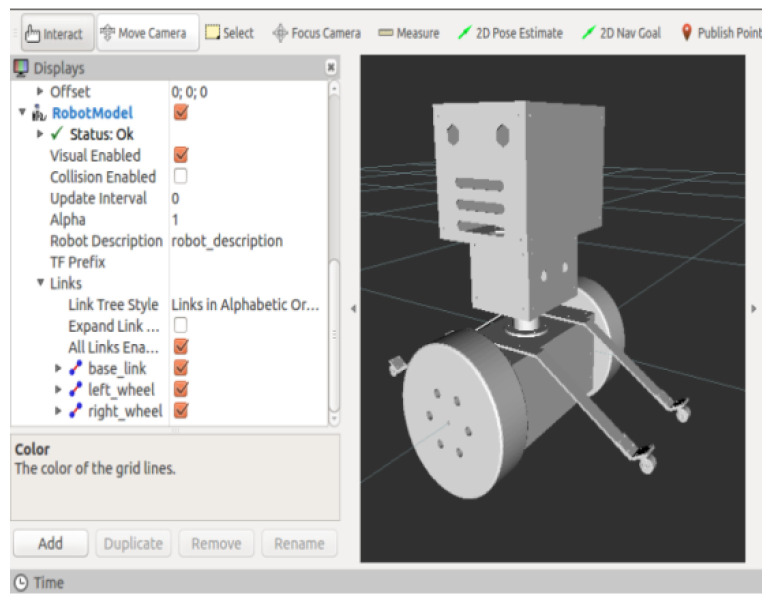
Inspection robot URDF model.

Firstly, a virtual simulation environment is built in Gazebo, and the map model is built by using the Building Editor model editing tool in the software bar. The two-dimensional plan is shown in Figure 16a. The map is designed as a double rectangular nested structure, with all walls being 1.5 m high and 0.15 m thick. The inner rectangular wall is 4 m long and 2 m wide; the outer rectangular wall is 8 m long and 6 m wide; a closed channel is formed between the inner wall and the outer wall to measure the environment space for the robot. In order to increase the difficulty of constructing the map, four additional rectangular walls were added at the midpoint of the four sides of the outer wall, with a wall length of 1 m and a wall width of 0.15 m. The 3D map model can be obtained through parameter design, as shown in Figure 16b. The URDF model of the inspection robot is shown in Figure 17.

**Figure 18:**
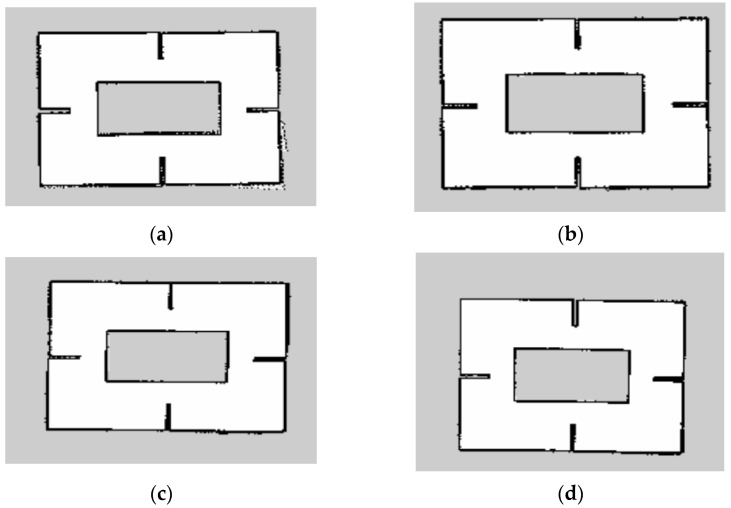
(**a**) The threshold for the number of particles is 20. (**b**) The threshold for the number of particles is 40. (**c**) The threshold for the number of particles is 60. (**d**) The threshold for the number of particles is 80.

The Gmapping algorithm is used to construct the environmental map. Since the Gmapping algorithm is based on the particle filter algorithm, the number of particles needs to be updated and iterated constantly in the process of constructing the environmental map. Therefore, it is of great significance to select appropriate particle parameters for constructing an accurate environmental map. In the process of the simulation experiment, the experimental variable is the number of particles, and different particle numbers are used to establish the map. Four groups of experiments are set in this paper, and the particle number threshold is set as 20, 40, 60, and 80, respectively. According to the selected particle number, we ran the Gmapping simulation algorithm to obtain the environment map model in Figure 18a–d and the measurement data table in Table 5, Table 6, Table 7, Table 8 and Table 9.

In the above simulation map, the color of the map represents different meanings, and the white represents the area that has proven to be free of static obstacles. The black color shows the static obstacle outline. It can be seen from the simulation results that, compared with the three-dimensional model, the closed channels and four prominent walls in the environmental map can be clearly displayed on the map, and the environment contour can be constructed using four different particles to construct the map. The contour of maps with particle number 20 in Figure 18a and 40 in Figure 18b shows different degrees of serrations. The map with particle number 60 in Figure 18c and 80 in Figure 18d shows relatively small places of serrations, and the map construction effect is good.

According to the simulation experiments of the Gmapping algorithm, compared with other SLAM algorithms, Gmapping effectively utilizes the wheel odometry information and can provide the pose priors of the robot [22]. There is no need for closed-loop processing in the operation process, so the computation is small. Through the simulation test, it is found that the local map is clear and of good quality and can be used as a navigation map. The indoor map can be constructed in real time, which requires less computation and higher accuracy in the construction of small scene map, and has low requirements on LiDAR frequency and good robustness.

## 5. The Experiment and Analysis of the Two-Wheel Self-Balancing Inspection Robot

### 5.1. Test and Analysis of the Robot Self-Balancing Algorithm

**Figure 19:**
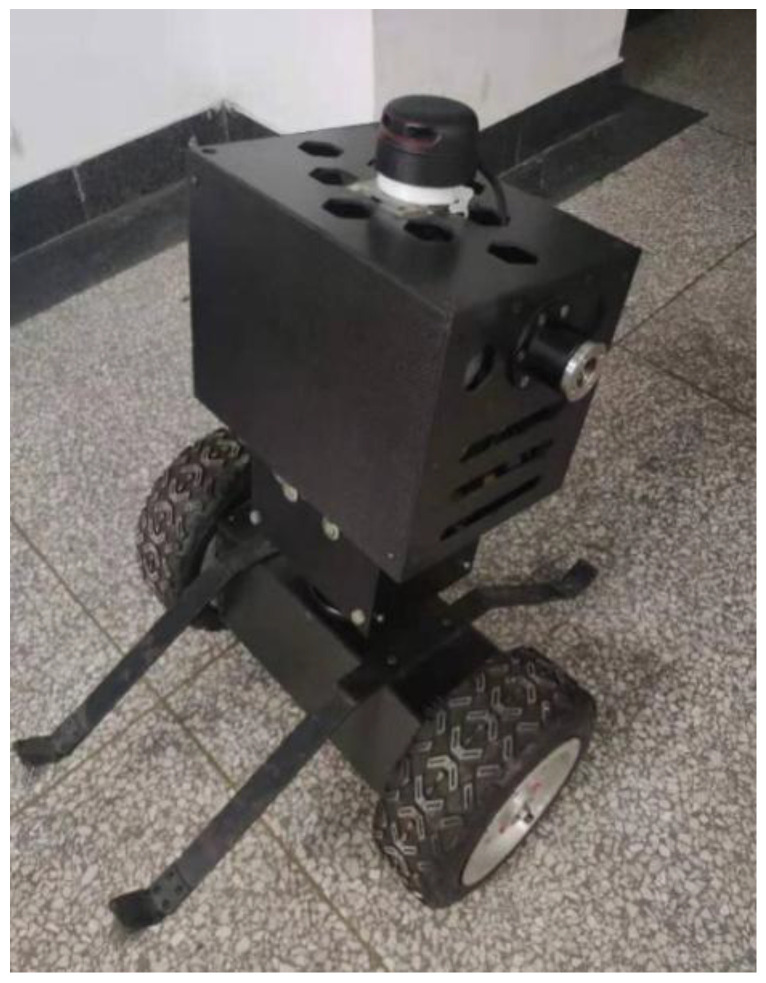
Physical map of inspection robot.

The mechanical mechanism of the inspection robot was processed and assembled, and the overall color was black. Electronic components were installed inside the structure, and electrical connections were made through wires. Finally, the program code was written, as shown in Figure 19 below.

In order to verify the robustness of the self-balancing algorithm designed in this paper, three groups of experiments are set, and the testing environment is the corridor on the seventh floor of the Science and Technology building. In the first set of experiments, the posture of the robot changed under the static state. In the second group of experiments, the posture of the robot changed in the self-balancing state. In the third group of experiments, the posture of the robot changed when a heavy load of 7.8 kg was applied.

#### 5.1.1. Pose Test of Robot at Rest

**Figure 20:**
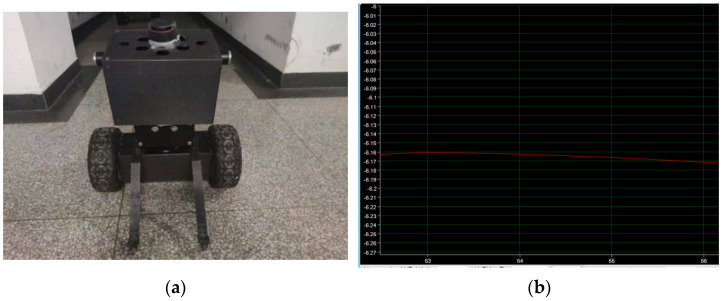
(**a**) Robot static experiment. (**b**) Waveform of robot at rest.

In the forward state of the robot, the auxiliary wheel of the protection support contacts with the ground to form a four-wheel stable structure, as shown in Figure 20a below. The STM32 controller collects the angle information of the gyroscope sensor and uploads the data to the terminal through the serial port to obtain the robot waveform diagram, as shown in Figure 20b below. As can be seen from the waveform curve in Figure 20b, when the robot is in the dumping state, the attitude output is stable, the attitude angle fluctuates around 6 degrees, and the data curve is relatively stable.

#### 5.1.2. Posture Experiment in a Self-Balancing Stable State

**Figure 21:**
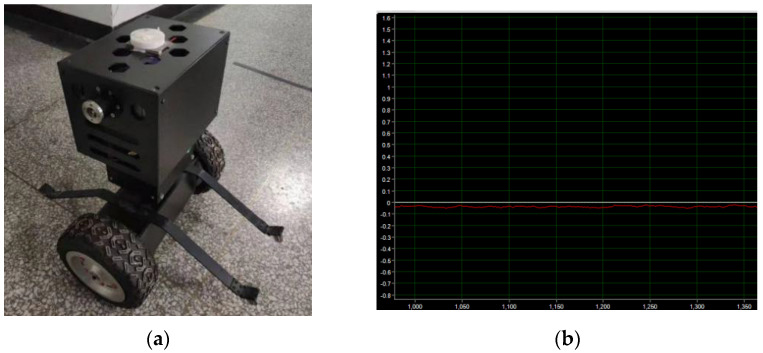
(**a**) Self-balancing experiment of inspection robot. (**b**) Self-balancing posture curve.

The self-balancing control of the two-wheel inspection robot is the precondition for the stable operation of the whole system. The inspection robot needs accurate and reliable angle information to maintain balance, which can be obtained by a gyroscope sensor. The self-balance experiment is shown in Figure 21a below. The angle deviation and angular velocity deviation are used to design the self-balancing control algorithm, which adopts proportional and differential control, that is, the traditional PD controller. After adjusting the parameters of the robot, the angular deviation ratio P parameter is set to 180, and the angular velocity differential D parameter is set to 0.78. It can be seen from Figure 21b that in the state of self-balancing control, the attitude curve fluctuates slightly up and down near the zero-degree position, and the robot maintains dynamic balance near the equilibrium point, with a certain anti-disturbance ability. When the robot is stable, there is a small static difference between the attitude curve and the zero-degree position. There are two main reasons for this phenomenon. The first reason is the accuracy of the algorithm. There will be errors in the measurement of the parameters of the established mathematical model, and the adjustment of the parameters of the algorithm model will also bring corresponding errors. The second reason is that the use of low-cost gyroscope sensors, sensor processing technology, and data acquisition process will also bring errors. However, under the control of the self-balancing algorithm, the static error phenomenon has relatively little influence on the robot body. When the robot is subjected to external interference, it still maintains a stable state, which indicates that the two-wheel mechanical structure of the inspection robot is reasonably designed and verifies the effectiveness and reliability of the balance control algorithm.

#### 5.1.3. Robot Attitude Experiment after Adding the Load

**Figure 22:**
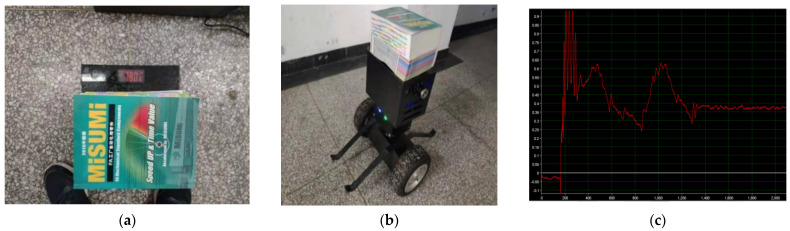
(**a**) Experimental load weighing. (**b**) Robot load experiment. (**c**) Waveform of robot attitude angle.

In order to verify the bearing capacity of the robot, in the design experiment, when the robot is in normal operation, a load of 7.8 kg is applied to the head of the robot, and the load mass is shown in Figure 22a below. The actual operation of the robot is observed, and the experimental figure is shown in Figure 22b. By collecting MPU 6050 sensor information, the robot attitude angle waveform is obtained, as shown in Figure 22c below. It can be seen from the attitude angle waveform diagram that the load experiment can be divided into three stages. In the first stage, before adding the load, namely 180 milliseconds, the waveform fluctuates in a small range around zero, and the robot is in a state of balance. In the second stage, a load of 7.8 kg was added at about 180 milliseconds, and the robot attitude curve jitter range was large. The robot in the equilibrium position needed to change its center of gravity in order to reach equilibrium again, the attitude curve began to fluctuate up and down, and the waveform gradually converged. In the third stage, after 1.3 s, the attitude curve completely converged and fluctuated in a small range of around 0.38 degrees. The attitude was in a stable state, and the robot reached a new equilibrium position. The deviation between the stabilized equilibrium position and the original equilibrium position was large, about 0.5 degrees. The main reason is that the load center of gravity is not on the center of gravity of the robot, and the robot needs to adjust the attitude angle to reach a new equilibrium point in order to reach a stable state. The experiment shows that the robot can reach the stable state without the initialization of the zero point, and has good adaptability and dynamic balance to the change of the load center of gravity. The robustness of the designed self-balancing algorithm is verified.

### 5.2. Map Construction Experiment of the Two-Wheel Self-Balancing Robot

**Figure 23:**
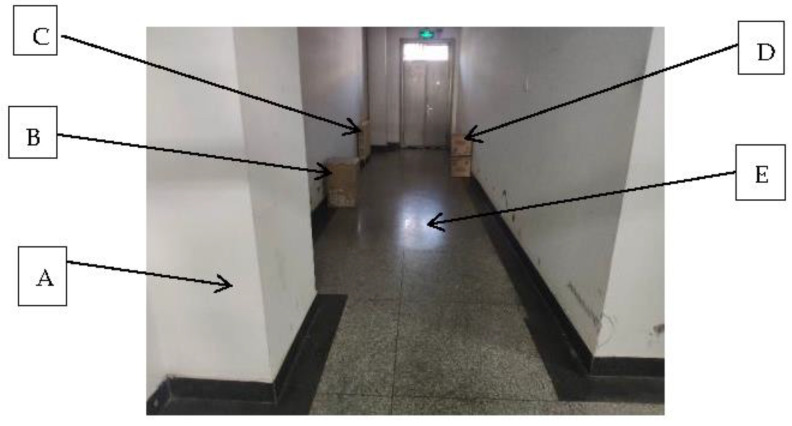
Actual test environment.

In this paper, the Gmapping SLAM algorithm based on particle filter theory is selected to realize the robot’s construction of an environment map in the ROS environment. The robot test environment is the corridor on the seventh floor of the Science and Technology Building, and all experiments are conducted indoors. The actual test environment is shown in Figure 23, where A, B, C and D are obstacles, and E is the width of the channel. In this section, two sets of experiments are set for the robot, and the threshold of particle number of the Gmapping algorithm is set to 60 and 80, respectively. The robot was run in the ROS environment, and the Gmapping algorithm was started to obtain the two-dimensional raster diagram of the environment.

**Figure 24:**
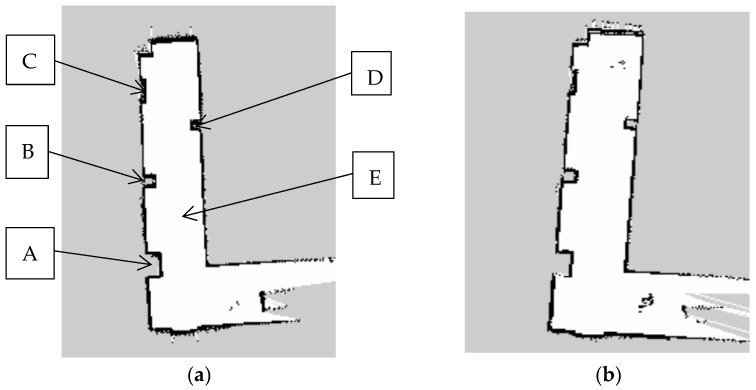
(**a**) Mapping effect with 60 particles. (**b**) Mapping effect with 80 particles.

The threshold of particle number was set to 60, and the environment map was constructed as shown in Figure 24a below, where A, B, C and D are obstacles, and E is the width of the channel. The threshold of particle number is set to 80, and the environment map is constructed as shown in Figure 24b below.

In the raster map, black represents areas with static obstacles, white represents areas without static obstacles, and gray represents unknown areas that have not yet been explored. A more complete map can be obtained by using different particle numbers in the algorithm. It can be seen that when the threshold of particle number is set to 60, the robot can construct a clear environment contour, and the map distortion is small. The threshold of particle number is 80. Although the robot can roughly build the environment map, the distortion of the map is obvious, and this phenomenon exists in both the prominent position in the lower left corner and the prominent position in the upper right corner. In order to verify the actual test accuracy of LiDAR, the data values of the four obstacles, A, B, C, and D, and the width of channel E in the two maps were measured, respectively, and then compared and analyzed with the real values during the construction of the map to calculate the absolute and relative errors, as shown in Table 10 and Table 11 below.

The experimental results show that when the threshold of particle number is set to 60, the relative error is 2.93%. When the threshold of particle number is set to 80, the relative error is 6.81%. It is known that the optimal threshold of particle number is 60. After the optimal value of particle number was determined to be 60, in order to test the mapping accuracy of the designed two-wheel self-balancing robot, the environmental mapping data with a load of 2.0 kg was tested in the laboratory corridor. Table 12 shows the following.

## 6. Discussion

This paper adopts the Gmapping algorithm for map construction. As can be seen from the data in Table 10, the relative error is 1.01%; as can be seen from the actual data in Table 12, the relative error is 2.93%. According to the data analysis, this paper adopts the Gmapping algorithm for map construction, and the accuracy of map construction is 1.97%. At the same time, it can be seen from the data analysis that, for example, the relative error is relatively large at the obstacle at point D. Especially when the load is 7.8 kg, the relative error is about 5.5%. The reason for this phenomenon is mainly because the load is too heavy and unstable, and map deviation will occur at the corner, affecting the accuracy of map construction. Since the two-wheel self-balancing robot designed in this paper is applied to the inspection of a downhole water pump room, carrying a load of about 2 kg, it can be seen from the experimental data in Table 12 that the drawing construction accuracy of the robot is suitable enough, so the inspection task can be successfully realized.

## 7. Conclusions

This paper designs and implements a laser SLAM-based inspection robot under the application background of safety inspection of the underground water pump room. The robot integrates environment perception, autonomous decision-making, and control execution, which provides a solution to many problems, such as mobile mode, functional requirements, and structural design of the traditional inspection equipment. As a nonlinear and underactuated unstable system, the two-wheel self-balancing structure simplifies the mechanical structure design and increases the difficulty of system control. The control mode is complicated, and the speed is stable while the attitude is balanced. However, the structure has the characteristics of long endurance time, small volume, flexible steering, and so on, and can be applied to a wide range. It is a good choice for the inspection of the underground water pump house. The double-closed-loop PID algorithm was used as the self-balancing control algorithm of the two-wheel robot, A2 LiDAR, was used as the SLAM environment sensing sensor, and combined with the Gmapping algorithm based on particle filter theory, multiple sets of data were selected to compare and obtain the most appropriate particle number for map construction. After the rationality of the system was verified by the simulation, the map construction of the corridor outside the experiment was completed in the actual test, and the inspection task was successfully realized.

## Figures and Tables

**Table 1:** 1023 Carbon steel material attribute table.

The Material Properties	The Parameter Value	Unit
Modulus of Elasticity	2.05 × 10^11^	N/m^2^
Poisson’s Ratio	2.9 × 10^−1^	None
The Shear Modulus	8 × 10^10^	N/m^2^
Mass Density	7.858 × 10^3^	kg/m^3^
The Tension Strength	4.25 × 10^8^	N/m^2^
The Yield Strength	2.82685 × 10^8^	N/m^2^

**Table 2:** Carbon fiber board material properties.

The Material Properties	The Parameter Value	Unit
Modulus of Elasticity	1.18 × 10^11^	N/m^2^
Poisson’s Ratio	3.35 × 10^−1^	None
The Shear Modulus	3.32 × 10^9^	N/m^2^
Mass Density	2.0 × 10^3^	kg/m^3^
The Tension Strength	3.0 × 10^7^	N/m^2^
The Yield Strength	1.5 × 10^8^	N/m^2^

**Table 3:** Head structure statics solution parameter table.

Name	Type	The Minimum Value	The Maximum Value
Stress	VON: von Mises	0 N/m^2^	1.29208 × 10^6^ N/m^2^
The Displacement	URES	0 mm	1.54699 ×10^−2^ mm
Strain	ESTRN	0	7.60008 × 10^−6^

**Table 4:** Bottom structure statics solution parameter table.

Name	Type	The Minimum Value	The Maximum Value
Stress	VON: von Mises	1.48067 × 10^3^ N/m^2^	4.2585 × 10^6^
The Displacement	URES	0 mm	1.35066 × 10^−2^ mm
Strain	ESTRN	1.27093 × 10^−8^	2.06188 × 10^−5^

**Table 5:** Measurement data with a particle count of 20.

Measurement Reference Point	Label	The Actual Value/cm	Measured Value/cm	Absolute Error/cm	The Relative Error
A	1	400	393.2	6.8	1.7%
B	2	200	204.4	4.4	2.2%
C	3	570	566.8	3.2	0.6%
D	4	100	101.1	1.1	1.1%
E	5	15	17.0	2	13.3%
F	6	770	766.6	3.4	0.4%
G	7	85	82.3	2.7	3.2%
H	8	185	181.4	3.6	1.9%
I	9	185	181.9	3.1	1.7%

**Table 6:** Measurement data with a particle count of 40.

Measurement Reference Point	Label	The Actual Value/cm	Measured Value/cm	Absolute Error/cm	The Relative Error
A	1	400	404.5	4.5	1.1%
B	2	200	198.8	1.2	2.2%
C	3	570	565.5	3.5	0.6%
D	4	100	96.5	3.5	1.1%
E	5	15	17.3	2.3	15.3%
F	6	770	766.1	3.9	0.5%
G	7	85	82.1	2.9	3.4%
H	8	185	182.9	2.1	1.1%
I	9	185	182.8	2.2	1.2%

**Table 7:** Measurement data with a particle count of 60.

Measurement Reference Point	Label	The Actual Value/cm	Measured Value/cm	Absolute Error/cm	The Relative Error
A	1	400	399.4	0.6	0.1%
B	2	200	199.2	0.8	0.4%
C	3	570	568.8	1.2	0.2%
D	4	100	101.5	1.5	1.5%
E	5	15	16.4	1.4	9.3%
F	6	770	764.9	5.1	0.7%
G	7	85	83.2	1.8	2.1%
H	8	185	183.8	1.2	0.6%
I	9	185	182.7	2.3	1.2%

**Table 8:** Measurement data with a particle count of 80.

Measurement Reference Point	Label	The Actual Value/cm	Measured Value/cm	Absolute Error/cm	The Relative Error
A	1	400	402.7	2.7	0.7%
B	2	200	204.3	4.3	2.2%
C	3	570	567.5	2.5	0.4%
D	4	100	103.1	3.1	3.1%
E	5	15	17.2	2.2	14.7%
F	6	770	767.1	2.9	0.4%
G	7	85	84.0	1.0	1.2%
H	8	185	183.3	1.7	0.9%

**Table 9:** Renewal.

Measurement Reference Point	Label	The Actual Value/cm	Measured Value/cm	Absolute Error/cm	The Relative Error
I	9	185	181.1	3.9	2.1%

**Table 10:** Measurement data with a particle count of 60 (with 7.8 kg load).

Reference Point	Label	The Actual Value/cm	Measured Value/cm	Absolute Error/cm	The Relative Error
The length of the obstacle at point A	1	84	85.2	1.2	1.43%
The width of the obstacle at point A	2	48	48.9	0.9	1.88%
The length of the obstacle at point B	3	44	45.4	1.4	3.18%
The width of the obstacle at point B	4	35	36.5	1.5	4.28%
The length of the obstacle at point C	5	92	90.3	1.7	1.85%
The width of the obstacle at point C	6	15	14.6	0.4	2.67%
The length of the obstacle at point D	7	32	33.7	1.7	5.31%
The width of the obstacle at point D	8	32	33.8	1.8	5.63%
Width of passage	9	204	204.5	0.5	0.25%

**Table 11:** Measurement data with a particle count of 80 (with 7.8 kg load).

Reference Point	Label	The Actual Value/cm	Measured Value/cm	Absolute Error/cm	The Relative Error
The length of the obstacle at point A	1	84	86.6	2.6	3.10%
The width of the obstacle at point A	2	48	49.4	1.4	2.91%
The length of the obstacle at point B	3	44	47.2	3.2	7.27%
The width of the obstacle at point B	4	35	38.8	3.8	10.9%
The length of the obstacle at point C	5	92	96.9	4.9	5.32%
The width of the obstacle at point C	6	15	16.4	1.4	9.33%
The length of the obstacle at point D	7	32	35.9	3.9	12.1%
The width of the obstacle at point D	8	32	35.1	3.1	9.69%
Width of passage	9	204	205.5	1.5	0.74%

**Table 12:** Measurement data with a particle count of 60 (with 2.0 kg load).

Reference Point	Label	The Actual Value/cm	Measured Value/cm	Absolute Error/cm	The Relative Error
The length of the obstacle at point A	1	84	84.3	0.3	0.36%
The width of the obstacle at point A	2	48	48.4	0.4	0.83%
The length of the obstacle at point B	3	44	44.6	0.6	1.36%
The width of the obstacle at point B	4	35	34.5	0.5	1.43%
The length of the obstacle at point C	5	92	91.3	0.7	0.76%
The width of the obstacle at point C	6	15	14.7	0.3	2.00%
The length of the obstacle at point D	7	32	32.9	0.9	2.81%
The width of the obstacle at point D	8	32	33.8	1.8	5.62%
Width of passage	9	204	204.5	0.5	0.25%

## Data Availability

The data are available upon request.
